# Galectin-3 and peripheral artery disease: a Mendelian randomization study

**DOI:** 10.3389/fcvm.2023.1279396

**Published:** 2024-01-04

**Authors:** Yang Gou, Miao Chen, Zhi Zhu, Chi Cui

**Affiliations:** Department of General Surgery, The Third People’s Hospital of Chengdu, Chengdu, Sichuan Province, China

**Keywords:** galectin-3, peripheral artery disease, Mendelian randomization, causality, SNPs (single-nucleotide polymorphisms)

## Abstract

**Background:**

Multiple clinical studies have found a significant correlation between elevated galectin-3 (Gal-3) in circulation and the diagnosis and severity of peripheral arterial disease (PAD). The current study used the Mendelian randomization (MR) technique to evaluate the possible causal relationship between Gal-3 and PAD.

**Methods:**

Genome-wide association study (GWAS) data of Gal-3 and PAD were obtained through the MR-Base platform. Then, using Gal-3 as the exposure and PAD as the outcome, a two-sample MR analysis was performed utilizing several regression techniques, including MR-Egger regression, inverse variance weighted (IVW), weighted median, and weighted mode.

**Results:**

Six single-nucleotide polymorphisms (SNPs) were identified and designated as instrumental variables (IVs) that exhibited significant correlations with Gal-3 (linkage disequilibrium *r*^2^ < 0.001; *P* < 5 × 10^−8^). Various statistical methods showed that there was an absence of a significant link between Gal-3 and PAD (IVW: odds ratio (OR) = 0.9869, 95% confidence interval (CI) = 0.8792–1.1078, *P* = 0.8232). In addition, the presence of genetic pleiotropy did impact the putative causal relationship between PAD and Gal-3 (MR-Egger intercept = 0.0099, *P* = 0.659).

**Conclusions:**

There is no current evidence to establish a causal relationship between the level of Gal-3 in circulation and PAD.

## Introduction

Peripheral artery disease (PAD) is a common cardiovascular disorder defined by the narrowing or occlusion of arteries in the peripheral arteries, resulting in diminished blood circulation to the lower limbs. It is a significant public health burden, affecting millions of individuals worldwide and causing substantial morbidity and mortality (1). Therefore, identifying novel biomarkers associated with PAD is of paramount importance for improving our understanding of the disease pathogenesis, enhancing risk stratification, and developing targeted interventions.

Galectin-3 (Gal-3) — a β-galactoside-binding lectin — has emerged as a promising biomarker in various cardiovascular disorders due to its involvement in inflammation, fibrosis, and vascular remodeling (2). It exerts multifaceted effects on different pathological processes, including cardiac remodeling, heart failure, and atherosclerosis (3). Moreover, Gal-3 has been implicated in the modulation of endothelial cell activity and angiogenesis and is closely correlated with the advancement and progression of PAD (4). No studies that show a correlation between the cellular levels of galectins and the levels of galectins in circulation.

Numerous clinical trials have found an association between elevated Gal-3 and the diagnosis and severity of PAD (5–9). However, these studies are largely observational, making it difficult to establish causality and rule out confounding factors, and currently, no studies have indicated a correlation between the levels of galectins in circulation and their cellular counterparts. Mendelian randomization (MR) is a powerful tool that overcomes limitations inherent in observational studies and provides more robust evidence for causal relationships (10).

The present study used the MR technique to evaluate the possible causal relationship between Gal-3 and PAD. This technique employs genetic variations as instrumental variables (IVs) to assess the causal influence of an exposure (Gal-3) on an outcome (PAD) (11). Genetic data derived from extensive genome-wide association studies (GWAS) were utilized to assess the potential association between genetically determined elevated Gal-3 and a heightened susceptibility to PAD.

## Methods

### Study overview

**Figure 1:**
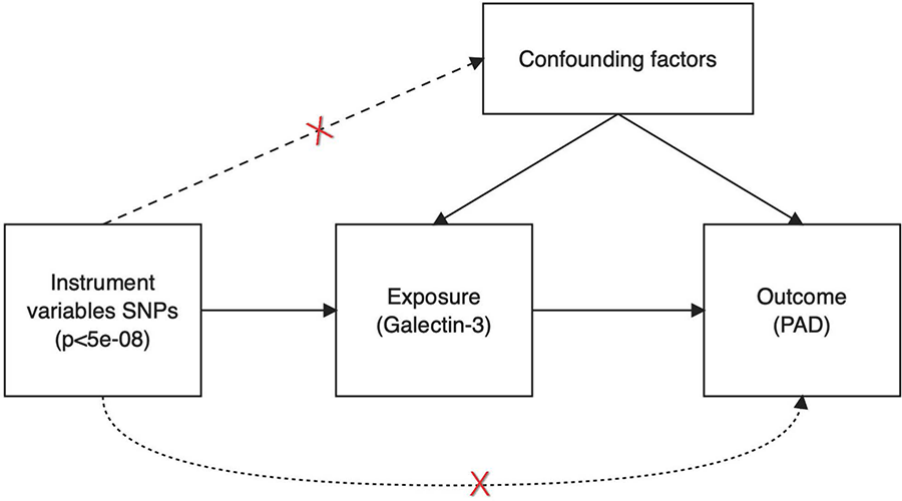
Schematic illustrating the underlying presumptions of Mendelian randomization analysis. SNPs, single nucleotide polymorphisms; PAD, peripheral artery disease.

The scheme of a two-sample MR analysis is displayed in Figure 1. Using single-nucleotide polymorphisms (SNPs) were applied as IVs and a two-sample MR strategy, the causal relationship between Gal-3 and PAD was examined. MR depends on three assumptions: (1) there is a significant association between IVs and Gal-3. (2) The presence of IVs does not exhibit any correlation with confounding variables in the relationship between the exposure variable and the outcome variable. (3) IVs alone influence the outcome via the mechanism of exposure and not through other pathways.

#### Data sources and selection of IVs

GWAS summary statistics for exposure (Gal-3) and outcome (PAD) were sourced from the MR-Base platform. The Gal-3 dataset includes 21,758 European ancestry individuals (12). The PAD dataset contains 212,453 individuals with East Asian ancestry, including 3,593 cases and 208,860 control groups. To mitigate the influence of linkage disequilibrium (LD) within a span of 10,000 kb, a genome-wide significance threshold was used (*P* < 5 × 10^−8^) and correlated variants with *r*^2^ < 0.001 were retained. Six SNPs were used as IVs. The F statistic is often used to assess the robustness of the association between IVs and exposure factors, specifically in the context of SNPs. To ensure the use of robust genetic instruments, the F statistic should exceed a threshold of 10 (13). F = (beta/se)^2^, where beta and se represent the effect size and standard error respectively.

#### Statistical analysis

MR analysis was performed in the MR-Base platform [R version: 4.0.3, App version: 1.4.3 8a77eb (October 25, 2020), R/TwoSampleMR version: 0.5.5] (14). The inverse variance weighted (IVW) technique was used to evaluate the causal relationship for each SNP by performing a meta-analysis of the Wald ratio that has been included in the study (15). However, the IVW approach presupposes the absence of pleiotropy in all instrumental variables. Provided that one or more SNPs do not adhere to the underlying assumptions of IVs, a bias will arise. Thus, when using the IVW method, IVs should not be pleiotropic (11). The MR-Egger analysis was applied to identify the presence of horizontal pleiotropy using its intercept test (16). The absence of pleiotropic effects is demonstrated if the intercept test of the MR-Egger method does not show a statistically significant difference from zero. The weighted median (WM) method was employed to assess the causal effects by permitting a maximum of 50% of the variables inside the SNPs to function as non-valid IVs (17). A significant potential causal relationship between Gal-3 and PAD was considered when the following three requirements were met. (1) The IVW method was significantly different (*P* < 0.05); (2) IVW, MR-Egger, and WM methods were consistent in their estimation directions; (3) There was no significance (*P* > 0.05) in the MR Egger intercept test.

## Results

### Gal-3 IVs

**Figure 2:**
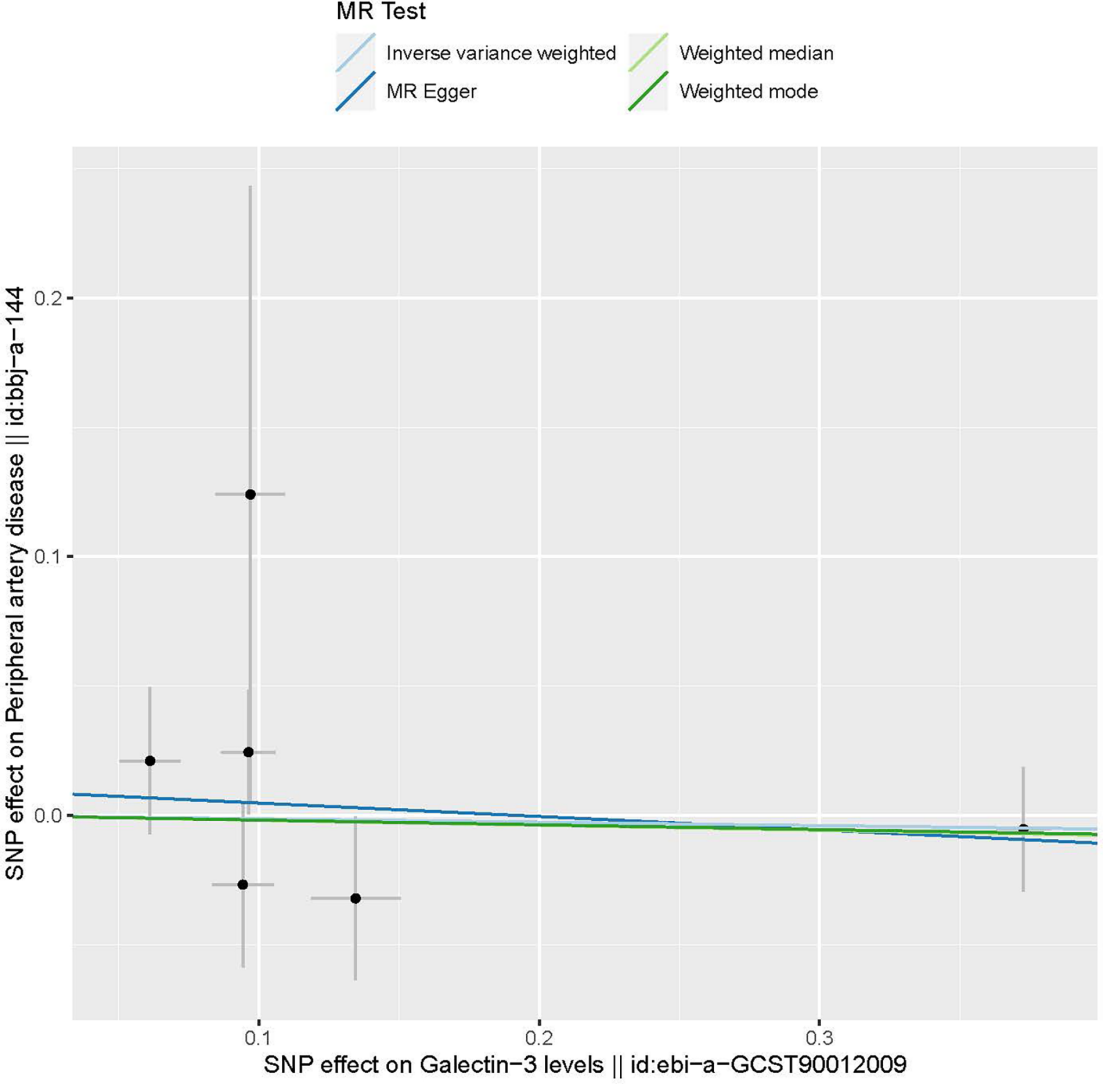
The provided scatter plot illustrates the potential causal association between galectin-3 and the risk of peripheral artery disease. The magnitude of the slope of the line is indicative of the degree of strength in the causal link. MR, Mendelian randomization; SNP, single nucleotide polymorphism.

**Figure 3:**
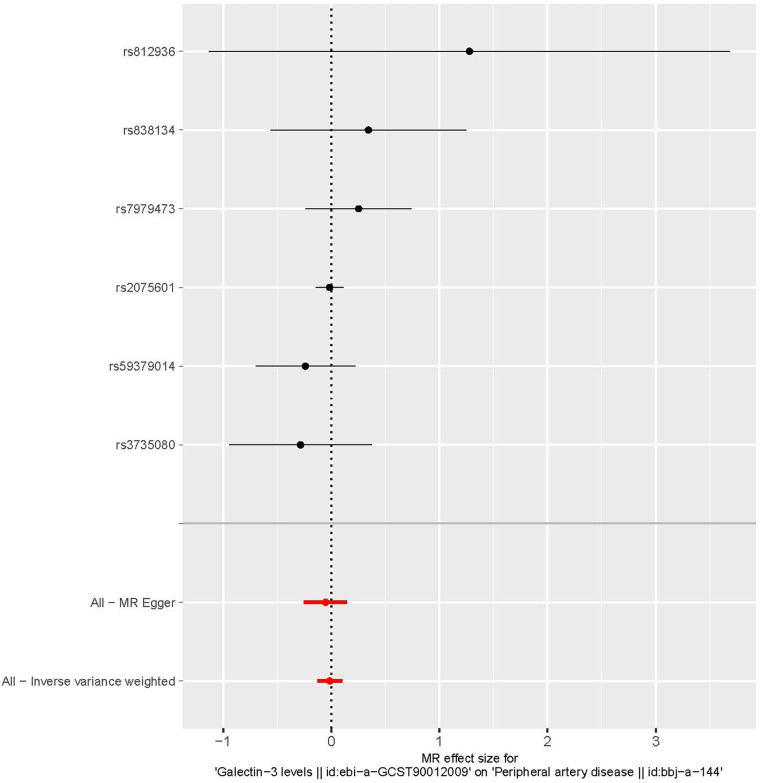
A forest plot illustrating the causative effects of single nucleotide polymorphisms (SNPs) that are connected with galectin-3 on peripheral artery disease. MR, Mendelian randomization.

The characteristics of the SNPs associated with Gal-3 and PAD are shown in Table 1. Six SNPs were finally selected as IVs (rs2075601, rs3735080, rs59379014, rs7979473, rs812936, and rs838134). All Gal-3-associated genetic instruments reached a genome-wide significance (*P* < 5 × 10^−8^). The F-statistic value ranged from 32.108 to 1,476.167 for the selected individual IVs. Thus, none of the SNPs were weak instruments. The causal effect of each genetic variation on PAD is depicted in Figures 2, 3. A potential causal relationship between exposure and outcome may exist, but it is not statistically significant.

**Table 1:** The features of SNPs linked to galectin-3 levels and investigates their potential correlations with peripheral artery disease.

SNP	Chr	Gene	EA	OA	EAF exposure	EAF outcome	Beta exposure	Beta outcome	SE exposure	SE outcome
rs2075601	14	LGALS3	T	C	0.4330	0.4635	−0.3727	0.0054	0.0097	0.0241
rs3735080	7	GIMAP7	T	C	0.2395	0.1733	0.0943	−0.0268	0.0109	0.0318
rs59379014	11	ST3GAL4	T	C	0.0771	0.1859	0.1345	−0.0321	0.0159	0.0315
rs7979473	12	HNF1A	G	A	0.6025	0.5271	−0.0963	−0.0244	0.0097	0.0241
rs812936	19	FUT3	A	G	0.8036	0.9898	0.0970	0.1240	0.0124	0.1191
rs838134	19	FUT1	C	A	0.2920	0.3319	−0.0612	−0.0211	0.0108	0.0283

SNP, single nucleotide polymorphism; Chr, chromosome; EA, effect allele; OA, other allele; EAF, effect allele frequency; SE, standard error.

### MR analysis

**Figure 4:**
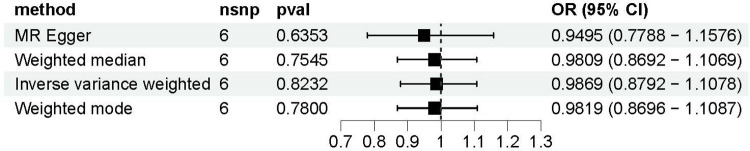
A forest plot to visually represent the causative impact of galectin-3 on peripheral artery disease. MR, Mendelian randomization; SNP, single nucleotide polymorphism; OR, odds ratio; CI, confidence interval.

The causal relationship between Gal-3 and PAD was examined using MR-Egger, IVW, weighted mode, and WM methods (Figure 4). The IVW approach revealed there was no statistically significant association between Gal-3 and PAD (odds ratio (OR) = 0.9869, 95% confidence interval (CI) = 0.8792–1.1078, *P* = 0.8232). Similar results were also observed for the other three methods (MR-Egger: OR = 0.9495, 95% CI = 0.7788–1.1576, *P* = 0.6353; WM: OR = 0.9809, 95% CI = 0.8692–1.1069, *P* = 0.7545; weighted mode: OR = 0.9819, 95% CI = 0.8696–1.1087, *P* = 0.7800).

### Sensitivity analysis

**Figure 5:**
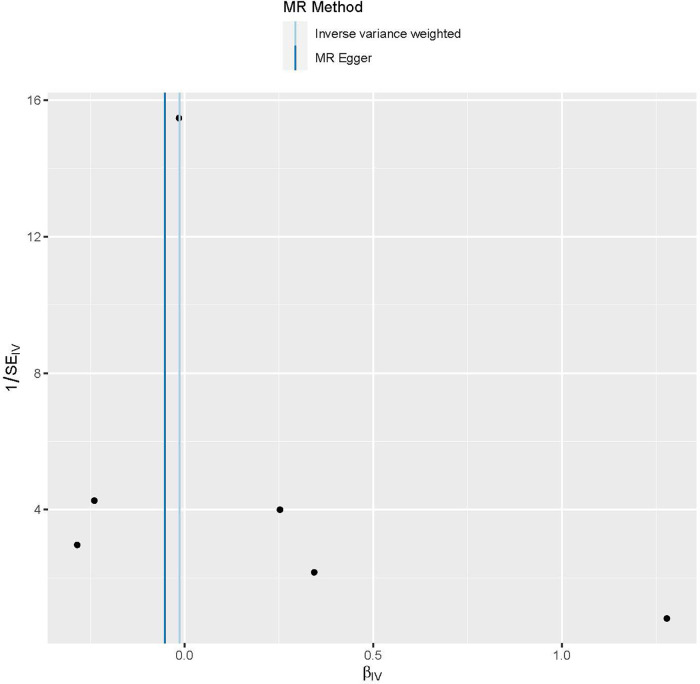
Funnel plots are used as a visual tool to depict the overall heterogeneity of the MR estimations pertaining to the impact of galectin-3 on peripheral artery disease. MR, Mendelian randomization.

**Figure 6:**
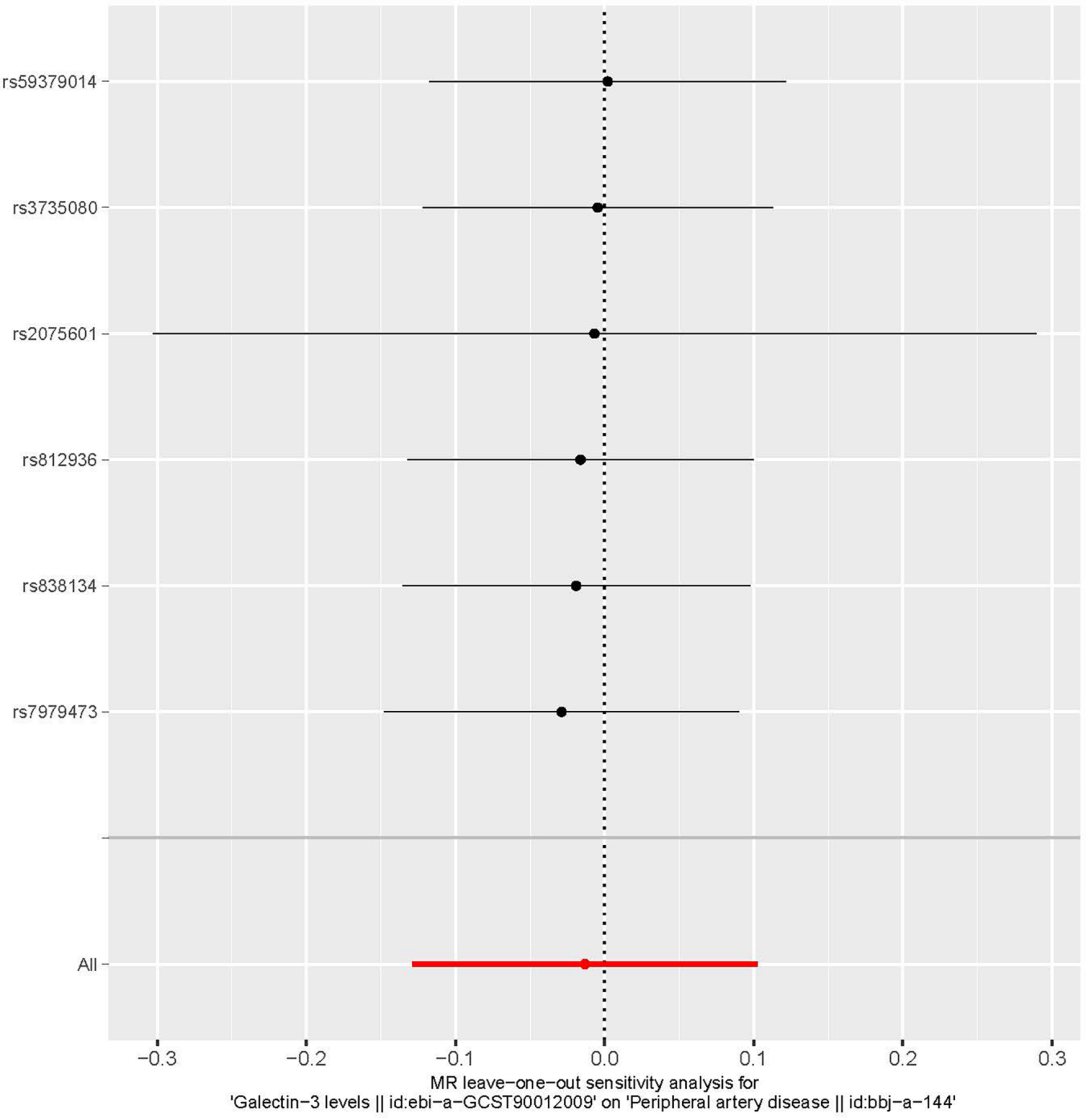
A leave-one-out plot is used as a visual tool to depict the causative influence of galectin-3 on the comprehensive risk associated with peripheral artery disease when one single nucleotide polymorphism is left out. MR, Mendelian randomization.

Funnel plots are a useful tool for visualizing the existence of horizontal pleiotropy in IVs. They can plot a single Wald ratio per SNP to display the directional level pleiotropy of the IVs. Detecting horizontal pleiotropy using funnel plots is challenging due to the limited inclusion of IVs. The funnel plot revealed a rough symmetry suggesting a potential for causality (Figure 5). Additionally, the absence of horizontal pleiotropy in the MR-Egger regression intercepts (*P* = 0.659) provided additional evidence that the phenomenon of pleiotropy did not create any kind of bias in the causal relationship. A leave-one-out approach was used to evaluate the individual influence of each SNP on the overall estimation of causation. The MR analysis was conducted systematically, whereby each of the SNPs was removed one at a time. This process was repeated on the remaining SNPs, as shown in Figure 6. The results showed a significant level of consistency, suggesting that the computed results for all SNPs demonstrated a statistically significant causal relationship. This finding suggests that there was no prominent SNP in the correlation between Gal-3 and PAD, therefore corroborating prior MR findings.

## Discussion

Based on current understanding, the link between Gal-3 and PAD was investigated in this first MR study. The hypothesis that Gal-3 increases the risk of PAD was not supported by the results of the present MR study, which is often considered to be less prone to confounding factors compared with observational studies. The relationship between Gal-3 and PAD has been studied in numerous observational studies. Ursli et al. (9) reported an association between Gal-3 and the severity of PAD. Their findings showed that there was an elevation in Gal-3 among individuals exhibiting symptoms of claudication, and this increase was found to be associated with a decrease in the ankle-brachial index. The presence of Gal-3 was also observable in urine samples, and its correlation with long-term mortality was observed in individuals diagnosed with PAD. However, their study had several limitations, including the assessment of Gal-3 in a specific group of individuals without critical limb ischemia and the quantification of Gal-3 in urine and serum only once, which limits the assessment of longitudinal values. Ding et al. (6) found that the presence of high-sensitivity C-reactive protein (hs-CRP) and Gal-3 in the general population was independently linked to the progression of PAD. This association provides evidence that fibrosis and inflammation significantly contribute to the pathophysiology of PAD. Inflammation assumes a significant role in the pathophysiology of cardiovascular disease, particularly PAD. They also found a positive relationship between the biomarkers Gal-3 and hs-CRP and their association with PAD and critical limb ischemia, regardless of the presence of other established atherosclerotic risk factors. The incorporation of Gal-3 and hs-CRP into conventional atherosclerotic predictors resulted in a slight improvement in the prognostication of PAD. Their study results suggest that Gal-3 and hs-CRP may be used as biomarkers to identify people who are at a higher risk for PAD and guide targeted screening and prevention efforts. However, their study is also limited in that it relied on data from the Atherosclerosis Risk in Communities (ARIC) study cohort, which included only participants aged 45–64 years. A different study revealed that there was a 22% increase in Gal-3 among individuals diagnosed with PAD, which was positively correlated with hs-CRP and homeostasis model assessment. However, the study did not adjust for other variables such as medication use, cardiovascular risk factors, ankle-brachial index, or biomarkers (5). Additional research is required to ascertain whether Gal-3 has a superior capacity compared with hs-CRP in serving as an indicator of heightened susceptibility to cardiovascular events. Madrigal-Matute et al. (8) inferred that Gal-3 holds promise as a possible biomarker for the detection and assessment of atherosclerosis, suggesting that inflammation, circulating cell infiltration, and oxidative stress participate in the underlying mechanisms of atherosclerosis. In addition, circulating Gal-3 concentrations were associated with clinical outcomes in patients with atherosclerosis. However, the study has some limitations, including a small sample size and failure to adjust for heart failure and coexisting cancers. Therefore, additional research is required to evaluate the therapeutic applicability of Gal-3 assays in predicting cardiovascular risk. Our findings are consistent with a recent study in which serum Gal-3 was not found to be a predictor of cardiovascular events and death in individuals with PAD but without significant limb ischemia (18). There is currently no evidence indicating a correlation between the level of Gal-3 in circulation and the level present in cell.

Our MR analysis has several notable strengths. First, this study represents the inaugural investigation into the causal relationship between Gal-3 and PAD using a two-sample MR methodology, leveraging a substantial volume of GWAS data. Secondly, similar to prospective randomized controlled trials, MR mitigates systemic biases affecting traditional observational studies, including confounding and reverse causality. Thirdly, the F-statistic was used to ensure that the IVs that were used were strong genetic tools. To evaluate the strength and reliability of the results, MR-Egger regression tests were used, which did not provide any indication of the presence of directional level pleiotropy.

Nonetheless, this study also has some limitations. First, we used aggregate data from the GWAS and thus could not perform subgroup analyses due to a dearth of precise data on sex and age. Second, Gal-3 data used in the current study were from individuals with European ancestry, whereas PAD data were from individuals with East Asian ancestry. Hence, the results may not be directly extrapolated to other populations, which may introduce bias due to population stratification and warrants further exploration. Finally, the OR was small. However, given the high prevalence of PAD, the focus on causality is justified.

## Conclusions

In summary, the current MR study found no evidence that the level of Gal-3 in circulation is associated with an elevated risk of PAD. The findings of the present study warrant further validation using larger sample sizes.

## Data Availability

Publicly available datasets were analyzed in this study. This data can be found here: https://gwas.mrcieu.ac.uk/.
